# Culture, Work, and Subjective Well-Being: The Role of LMX and Resilience in Spanish and Chinese Cultures

**DOI:** 10.3390/ijerph16244945

**Published:** 2019-12-06

**Authors:** Pilar González-Navarro, Elena Talavera-Escribano, Rosario Zurriaga-Lloréns, Lucía I. Llinares-Insa

**Affiliations:** 1 Instituto de Investigación en Psicología de los Recursos Humanos, del Desarrollo Organizacional y de la Calidad de Vida Laboral (IDOCAL), Universitat de València, 46010 Valencia, Spain; gonznava@uv.es (P.G.-N.); Rosario.zurriaga@uv.es (R.Z.-L.); 2 Centre for Applied Linguistics, University of Warwick, Coventry CV4 7AL, UK; elena.talavera-escribano@warwick.ac.uk; 3 Facultat de Psicologia, Universitat de València, 46010 Valencia, Spain

**Keywords:** leader-member exchange, culture, well-being, engagement, resilience

## Abstract

Globalization and interdependencies among nations require a better understanding of the influence of culture on organizational processes. In order to succeed in global business, leaders have to respond to practices that may be different in diverse cultures. This study was conducted within the framework of the leader member exchange approach and from a positive perspective of organizations linking successful businesses and workers’ well-being. The aim of this study was to examine whether the quality of the relationship with the leader predicts engagement and life satisfaction, and whether resilience moderates this relationship in two different cultural contexts (Spanish and Chinese). The sample was composed of 277 workers (127 Chinese workers corresponding to a vertical-collectivistic culture and 150 Spanish workers representing a horizontal-individualistic culture). To test the hypotheses, a structural equations model (SEM) was conducted using the maximum likelihood (ML) estimation method. Results revealed that leader-member exchange (LMX) positively predicts engagement and life satisfaction and that the moderator role of resilience varies across cultures. Resilience moderated the relationship between LMX and engagement and life satisfaction only in the Spanish sample. In the Chinese sample, resilience only moderated the relation between LMX and life satisfaction. Finally, our study contributes to a better understanding of the relationship between leaders and subordinates operating in a global context.

## 1. Introduction

The current increase in globalization and the constant interdependence among different nations require cross-cultural cooperation and a better understanding of leadership practices in different cultures. In order to succeed in global business, leaders have to be flexible enough to respond to practices that may be quite different from the ones to which they are accustomed [1]. People from different cultures differ in the way they value objects, ideas, or events [2]. In this research, we aimed to study leadership in two different cultural contexts, Spanish and Chinese, using the theoretical framework of leader member exchange (LMX). According to the LMX theory, leaders are closer, friendlier, more inclusive, and more communicative with some members who report to them than with others. In other words, leaders establish a relationship of high quality, trust, affection, and respect with some employees, whereas with others they have a lower-quality exchange that is limited to the employee’s and leader’s job descriptions regarding relations with other members [3].

Research has shown that leadership has an important role in the learning facet of employee resilience [4,5]. Resilience is defined as “the capacity to rebound from adversity strengthened and more resourceful” [6] (p. 97). In fact, resilience is a psychological coping mechanism to adapt to challenging work circumstances [7,8]. In this regard, there is a general consensus about the critical contribution of resilient employees to improving engagement, allowing them to survive major crises and improve when faced with challenges [5]. Nevertheless, considering the attention positive psychology has received in the past decade, little research has been dedicated to studying the relationship between LMX and positive psychology-related outcomes. Bauer and Erdogan [3] (p. 150) propose that “future research on LMX should also expand the lens to include broader concepts of satisfaction and well-being”. In fact, research on leadership behavior has mainly focused on the analysis of its effects on performance and, secondarily, on well-being, even though well-being should be “an important outcome in and of itself” [9] (p. 179). This represents a gap in the research on LMX. Therefore, the present study focuses on two positive outcomes contextualized in work and non-work domains: Engagement (defined as “energy that is directed toward organizational goals” [10] (p. 265)) and life satisfaction (defined as a “global evaluation by the person of his or her life” [11] (p. 113)). In addition, the differences in the quality of the relationship with the leader could be buffered by individual factors, such as resilience. Thus, resilience could help to understand the association between low LMX and engagement and life satisfaction. However, studies addressing this issue are scarce. Our study attempts to contribute to the literature by studying both personal and contextual factors simultaneously.

Furthermore, our study aims to advance the understanding of the role of LMX and resilience in engagement and life satisfaction in two different cultural contexts. We focus on this process because most of the leadership research has been conducted in a specific context: North America [12,13,14]. In fact, there is a call for further research focusing on the role of culture, in order to better understand LMX and its outcomes [15,16,17,18]. The consideration of cultural aspects has consequences, not only in the research field, but also in the practices organizations carry out.

Therefore, the goal of this study was to examine whether the quality of the relationship with the leader predicts engagement and life satisfaction, and whether resilience moderates this relationship in two cultural contexts (Spanish and Chinese).

Our study attempts to make several contributions to the literature. At a theoretical level, we provide information about the relationship between LMX and positive psychology-related outcomes contextualized in work and non-work domains. Moreover, we contribute to understanding how leadership acts in two different cultural contexts. At a practical level, our study provides a greater understanding of the relationship between leaders and subordinates operating in a global context.

### 1.1. LMX, Work Engagement and Well-Being

The quality of the relationship between leaders and followers is key to understanding leaders’ effects on members, teams, and organizations. In this regard, LMX is crucial in effective organizations, and several meta-analyses have shown that LMX has a positive impact on many organizational outcomes [18,19]. However, although high-quality LMX is known to have a positive impact on job-related well-being, few studies have investigated LMX as a predictor of individuals’ overall well-being [9].

Currently, the work–life interface is gaining importance in people’s well-being, due to the thin line that technology and new ways of working have drawn between work and personal life. As Day [1] pointed out, the study of the impact of LMX on the work–life balance is one of the most important future lines of study. Therefore, in this research, we aimed to study work/non-work domains in order to contribute to the LMX literature from a positive framework.

Positive psychology has received considerable attention from researchers because it makes it possible to enhance and promote healthy organizations [20]. This approach is concerned with well-being (e.g., life satisfaction) and the optimal functioning of individual outcomes in the organization (e.g., engagement) [21]. Research has indicated that when people develop and use their strengths, positive psychological and behavioral outcomes and higher levels of energy and vitality can be achieved [22,23].

On the one hand, engaged workers are more likely to be willing to devote effort and dedication to their work, in addition to showing enthusiasm, inspiration, and concentration on the job. By contrast, disengaged employees are more likely to be less involved at work, and they have a strong emotional disconnection with co-workers [24]. Likewise, high-quality leader–member exchange relationships have been found to enhance followers’ work engagement [25,26,27].

On the other hand, life satisfaction is one of the major indicators of subjective well-being [28]. This concept has been studied as an assessment of life, based on the fit and balance between personal goals and achievements [29,30] and a dimension of mental health [31]. Life satisfaction is determined by evaluating the satisfaction derived from various life domains, including work, family, and leisure activities. It is necessary to have satisfaction in the distinct domains in order to experience satisfaction with life, although people give different weights to each domain [32]. In fact, the quality of working life has been shown to have a positive effect on overall life satisfaction [33], and Kacmar, Carlson, and Brymer [34] found that LMX and life satisfaction were significantly correlated.

Based on the arguments presented above, we argue that when the leader has a high-quality relationship with the employees, they can develop and use their strengths and, thus, achieve higher engagement (work outcome) and life satisfaction (non-work domains). Therefore, we hypothesize the following:

**Hypothesis** **1.** The quality of the relationship between leaders and followers will be positively related to subordinates’ engagement and life satisfaction.

### 1.2. The Moderator Role of Resilience

Today, we live in a world where the scenarios change constantly, and the challenges are sometimes difficult and intense. Resilient people are able to use their skills and strengths to cope with and recover from the problems and challenges life presents. In fact, individuals with high levels of resilience perceive life challenges from a positive perspective [35].

Resilience has emerged as an important construct in organizations because organizations can get the most from their employees by focusing on positive psychological resources, such as resilience [36], especially in turbulent times [37]. In this regard, research has shown that resilience is positively correlated with engagement [38,39] and life satisfaction [40,41,42].

It is well known that work demands make it difficult for the leader to have the same quality in his/her exchanges with all subordinates. Thus, employees must have personal resources to buffer the effects of a low-quality relationship with the leader. Therefore, the combination of personal and external factors (e.g., resilience and LMX) allows employees to have a positive way of facing adversities [43] and, consequently, achieve positive outcomes. In the current highly competitive environment, it is necessary to understand the processes underlying the relationship between LMX and work and non-work domains. We propose that resilience acts as a moderator between LMX and work/non-work domains. Specifically, we seek to capture the role of resilience and its influence on the relationship between the quality of LMX and employees’ engagement and life satisfaction. From a practical perspective, understanding the mechanisms connecting these variables can be useful for designing strategies to manage work demands. Therefore, we hypothesize the following:

**Hypothesis** **2.** Resilience will moderate the relationship between LMX and engagement and life satisfaction, such that the relationship will be stronger when resilience is high rather than low.

### 1.3. Cross-Cultural Differences

Culture has been defined by Hofstede [13] (p. 9) as: “The collective programming of the mind that distinguishes the members of one group or category of people from another”. There are two main theoretical approaches in examining the effects of national culture. The conventional approach treats individual cultural value dimensions as predictors, whereas the configurational approach [44] focuses on configurations of cultural values. It is especially appropriate to study culture at a national level of analysis because societal cultural values are likely to co-occur.

The cultural approach distinguishes between horizontal individualism/vertical collectivism configurations [45,46,47]. The individualism–collectivism (IC) dimension is generally considered the most important cultural dimension for describing the effects of a society’s culture on the values, cognition, behavior, and social relationships of its members [48]. On the one hand, collectivist cultures foster the development of an interdependent self. Thus, people are seen as fundamentally interconnected with others close to them, and they emphasize the proper functioning of these relationships over their own goals. On the other hand, individualistic cultures encourage people to develop an independent sense of self and develop their own goals, motivations, and personality [46,49,50]. In other words, people in horizontal-individualistic cultures are more likely to regard themselves as independent from and equal in status to others, and this configuration is mainly found in Western societies. By contrast, people in vertical-collectivistic cultures are more likely to describe themselves as interdependent with others, and they hold greater respect for authority. These cultures are more representative of Asia. Paulienë [51] indicates that effective leadership in individualistic cultures focuses on the results derived from the behavior of the leader, whereas in collectivist societies, effective leadership is a lasting objective, and there is a dependence on leaders, who provide security and direction. Taras, Kirkman, and Steel [52] found that individualism–collectivism and power distance were the strongest predictors of a range of outcomes at the societal level. In turn, these values are strongly related to cross-cultural leadership in general [53,54] and LMX in particular [15,17].

As Rockstuhl et al. [17] state the values of horizontal individualism/vertical collectivism have direct implications for the relationship between LMX and organizational outcomes. LMX theory states that a main cause of positive organizational outcomes is high-quality LMX. Thus, strong relationships between LMX and outcomes have been found in nations with horizontal-individualistic cultures. In fact, comparative cross-cultural studies and meta-analyses on LMX [19] have indicated that the relationship between LMX and other variables (e.g., task performance, organizational citizenship behavior, perception of justice, job satisfaction commitment, and turnover intentions) may partly be culturally dependent.

A large number of studies on leadership follow “North American research templates”, an issue referred to by Tung [55] as “diverse homogeneity”, which can create an American bias in current leadership theories. As Solomon and Steyn [56] point out, leaders who do not take culture into account, especially during intercultural interactions, will be less effective. By contrast, leaders who are culturally aware and act accordingly will be increasingly effective.

Studies in this field [57,58,59] suggest that work–life research could be further expanded to include cross-cultural comparisons, due to the importance of this topic in the current globalized world. In addition, work engagement is embraced to a greater extent in individualistic cultures because individuals are more focused on achieving their personal goals, whereas in collectivist cultures, individuals are more concerned with group achievements [27,60,61]. Moreover, according to Veenhoven [62], culture plays an important role in explaining the factors that influence life satisfaction. In this regard, Erdogan, Bauer, Truxillo, and Masfield [63] find that work context characteristics are related to life satisfaction, particularly in individualistic nations.

Several studies state that resilience is more present in collectivistic cultures [64,65], especially in China, whose inhabitants easily develop some resilience strategies in unfavorable conditions [66,67]. As Wang et al. [43] state, culture plays an important role in the development of resilience, and so further research is necessary to clarify the role of culture and the environment and how they interact with individual traits to develop resilience.

**Figure 1:**
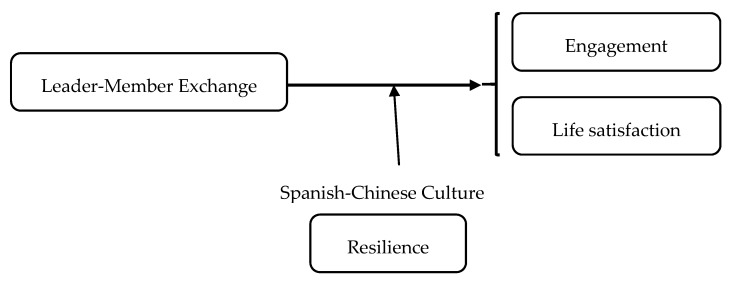
Hypothesized conceptual model.

The present study aimed to examine whether LMX predicts engagement and life satisfaction, and whether resilience moderates this relationship in two cultural contexts: Asian and European cultures (See Figure 1).

To do so, we selected a Spanish sample of workers (individualistic culture) and a Chinese sample of workers (collectivistic culture). In nations with horizontal-individualistic cultures, good quality relationships with the leader make it easier to achieve individual goals. The achievement of these goals may be associated with greater engagement and satisfaction with life. In nations with a vertical-collectivistic culture, interpersonal relationships are more important, and there is greater respect for authority. For this reason, interdependence with the leader is not as relevant in achieving engagement and satisfaction with life. Along with external factors, such as LMX quality, personal resources, such as resilience, can be useful for employees in facing adversities. Thus, resilience can be a moderator in this model of the relationship between LMX and work and non-work outcomes. Therefore, we hypothesized:

**Hypothesis** **3.** Resilience will moderate the relationships between leader–member exchange and engagement and life satisfaction in Spanish employees. These relationships will be higher in employees with high resilience. In Chinese employees, these relationships will be attenuated or non-significant.

## 2. Materials and Methods

### 2.1. Sample and Procedure

The study design was cross-sectional. The study sample was composed of Spanish and Chinese workers, corresponding to horizontal-individualistic and vertical-collectivistic national cultures, respectively, according to Hofstede’s dimensions [68]. The importance of taking these countries into account stems from the fact that, outside the European Union (EU), China is Spain’s largest trade partner, and Spain is one of China’s most important trade partners within the EU.

The total sample was composed of 277 workers (53.4% men and 46.6% women) with a mean age of 37.33 years (SD = 11.34).

The Spanish sample was composed of 150 workers (50.7% men and 49.3% women) with a mean age of 37.5 years (SD = 10.86). More than half of the sample work in the private sector (54.7%), and public sector employees make up the rest of the sample (45.3%). More than half of the sample are permanent workers (54.0%), whereas 46.0% are temporary workers. The respondents belong to different professional sectors, based on the classification of the Spanish Ministry of Labor and Immigration: Cultural and communal services (18.7%); administration and management (17.3%); trade and marketing (12%); hotel and tourism (4%); and other sectors, such as installation and maintenance; computers and communications; transport and maintenance of vehicles, etc. (26%), with missing values for 22%.

The Chinese sample was composed of 127 Chinese workers (56.7% men and 43.3% women) with a mean age of 39.3 years (SD = 11.23). Most the sample work in the private sector (96.9%), and public sector employees make up the rest of the sample (3.1%). Almost half of the sample are permanent workers (40.9%), whereas 59.1% are temporary workers. The respondents belong to different professional sectors, based on the classification of the Spanish Ministry of Labor and Immigration: Administration and management (40.6%); trade and marketing (18%); computers and communications (15.6%); installation and maintenance (9.4%); and other sectors, such as hotel and tourism; electrical and electronics; building and civil engineering; health, etc. (9.4%), with missing values for 7%.

The sampling procedure used was incidental purposive sampling [69]. We used this sampling technique because in this research, it was necessary to select employees working in two different cultures. Participants in the study had to be currently working. Another selection criterion, only for the Chinese participants, was that these workers had to be proficient in reading English. The first contact with the participants was made via email and social media networks (especially LinkedIn) to explain the aims and procedure of the study. Data were collected from 2016 to 2017 through self-report questionnaires completed voluntarily by the participants on-line, after they had provided their informed consent. The researchers stressed that anonymity and confidentiality were guaranteed, that there were no right or wrong answers, and that participants should answer the questions as honestly as possible [70].

### 2.2. Measures

For the Chinese sample, the questionnaires administered were in English. For the Spanish sample, the adapted and cross-validated Spanish version of each instrument was used. The following questionnaires were employed.

Leader Member Exchange. The quality of the leader–member relationship was measured using the Leader Member Exchange 7 Questionnaire (LMX-7) developed by Scandura and Graen [71]. This scale consists of seven items rated on a four-point Likert-type scale (1: Rarely to 5: Very often). High scores indicate high LMX quality, which means that the relationship is based on mutual trust, respect, satisfaction, understanding, and obligation between supervisors and employees. An example item is: “Do you usually know how satisfied your leader is with what you do?”. The Cronbach α coefficient for this scale was 0.88 in this sample.

Engagement. Engagement was measured using the short version of the Utrecht Work Engagement Scale (UWES) [72]. This scale has nine items rated on a 6-point Likert-type scale, ranging from 1 (almost never) to 6 (always). High scores indicate that the person is full of energy in her/his job, is happy when working intensely because s/he is proud of the work that s/he does, and feels like going to work every morning. An example item is: “At my work, I feel bursting with energy”. Cronbach’s alpha coefficient for this scale was 0.94 in this sample.

Satisfaction with life. Life satisfaction was measured using The Satisfaction with Life Scale (SWLS) developed by Useche and Sege [73]. This scale has five items rated on a 5-point Likert-scale, ranging from 1 (strongly disagree) to 5 (strongly agree). High scores indicate that the person feels that his/her life conditions are excellent, is satisfied with her/his life, and thinks that, so far, s/he has achieved the important things in life. An example item is: “In most ways, my life is close to my ideal”. Cronbach’s α coefficient for this scale was 0.84 in this sample.

Resilience. Resilience was measured using the Ego Resiliency Scale (ER-89) developed by Block and Kremen [74]. This scale consists of 10 items rated on a 6-point Likert-type scale, ranging from 1 (almost never) to 6 (always). High scores indicate that the person quickly recovers from being startled, is regarded as a very energetic person, gets over her/his anger at someone reasonably quickly, and would be willing to describe him/herself as having a pretty “strong” personality. An example item is: “I quickly get over and recover from being startled”. Cronbach’s α coefficient for this scale was 0.82 in this sample.

### 2.3. Data Analysis

Initial descriptive statistics and bivariate correlations between all the variables were examined. To test the hypotheses, a structural equation model (SEM) was conducted using the maximum likelihood (ML) estimation method. We tested a model that proposed the moderating effect of resilience in the relationship between LMX and engagement and life satisfaction.

A variable was created with LMX and resilience together as a moderating variable [75]. First, we tested the hypothesized relationship using structural equation modelling (SEM) in the total sample. Second, multi-group SEM was used to test the model in Spanish and Chinese employees. Third, SEM was carried out in Spanish and Chinese employees separately. The model was tested using the bias-corrected bootstrap confidence interval method [76]. Then, 5000 bootstrap resampling was performed with confidence intervals set at 95%. Maximum likelihood (ML) parameter estimates were calculated. Model fit was assessed with a combination of fit indices [77]. A nonsignificant chi square indicates good model fit; however, chi square is sensitive to sample size [78]. We also assessed the root mean square error of approximation (RMSEA), the comparative fit index (CFI), the normed fit index NFI, and the incremental fit index (IFI) with the conventional cut-off values (i.e., RMSEA < 0.08; CFI > 0.90; NFI > 0.90; IFI > 0.90 [79]. These fit values would indicate that the hypothesized relations in the model are plausible. If the initial model offers a poor fit to the data, the second step is to modify the model.

Combinations of descriptive and inferential statistics were calculated with the Statistical Package for the Social Sciences (SPSS), version 24 (IBM Corp., Armonk, NY, USA), and structural equation modelling (SEM) was performed using Analysis of Moment Structures (AMOS), version 24 (IBM Corp., Armonk, NY, USA). Sample size was adequate, correlations between variables were not high, 0.85 [80], and the sample followed a standard normal distribution [79].

## 3. Results

### 3.1. Descriptive and Preliminary Analysis

Means and standard deviations for the studied variables were calculated in the total sample, as well as Pearson bivariate correlations (Table 1). There were significant positive correlations between resilience and engagement, life satisfaction, and LMX (correlations between r = 0.37 and r = 0.65 ρ = 0.001).

### 3.2. Test of the Hypothesized Model

First, we tested the hypothesized model in the total sample. Table 2 presents the goodness of fit indices. The model presented an acceptable fit to the data. The estimators were significant in the total sample; thus, these results provide support for hypothesis 1: LMX positively predicts engagement and life satisfaction. The hypothesized moderating role of resilience in the relationship between LMX and engagement and life satisfaction (H2) was tested, and the results supported it. However, the estimator of resilience showed that this model could be improved because the relationship between resilience and engagement was not significant. Then, we analyzed the model in the Chinese and Spanish samples separately. The multi-group analysis (Table 2) showed an acceptable fit to the data, but the RMSEA was higher than in the total sample. The estimators were statistically significant in the Spanish sample (Table 3), but the estimator between LMX and engagement was not statistically significant in the Chinese sample. Therefore, the hypothesized model was tested in the Spanish sample and re-specified for the Chinese sample.

First, in the Spanish sample, the results showed that resilience moderates the relationships between LMX and engagement and life satisfaction (Table 3). This result supports our hypothesis 3: Resilience moderates the relationship between leader–member exchange and engagement and life satisfaction in Spanish employees.

**Figure 2:**
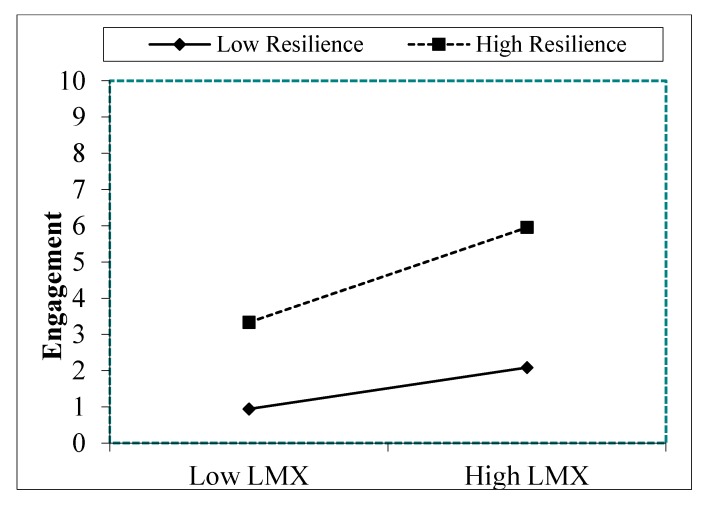
Interaction effects of resilience and LMX on engagement Note. LMX = Leader Member Exchange.

**Figure 3:**
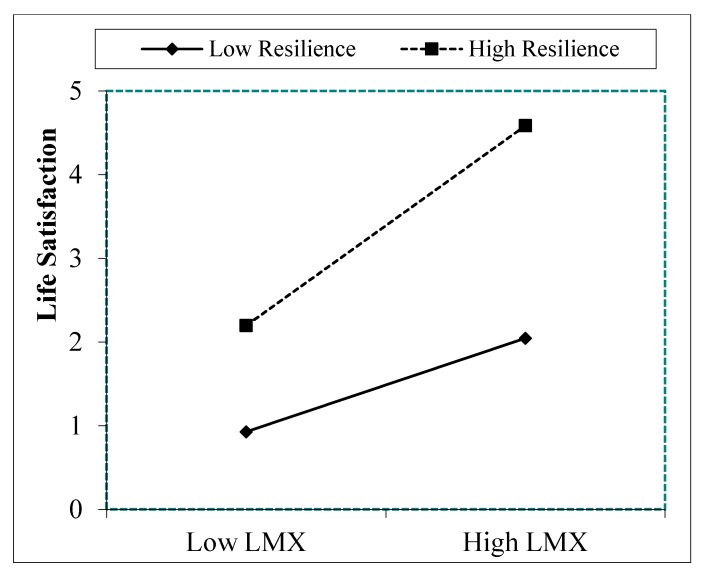
Interaction effects of resilience and LMX on life satisfaction Note. LMX = Leader Member Exchange.

To further interpret the interaction effect proposed in hypothesis 3, and based on Aiken and West [81] and Dawson [82], we computed simple slopes for high and low values of the moderator. Figure 2 and Figure 3 show that higher levels of LMX were associated with higher levels of engagement and life satisfaction when resilience was high. Low levels of LMX were associated with low levels of engagement and life satisfaction when resilience was low. These results show that the slopes for high resilience (Engagement: *t* = 0.11; *p* = 0.001; Life Satisfaction: *t* = 0.10; *p* = 0.001) and low resilience (Engagement: *t* = 0.16; *p* = 0.001; Life Satisfaction: *t* = 0.10; *p* = 0.001) were significant. Moreover, Figure 2 and Figure 3 reveal that engagement and life satisfaction were always lower in the presence of low resilience than in the presence of high resilience.

**Figure 4:**
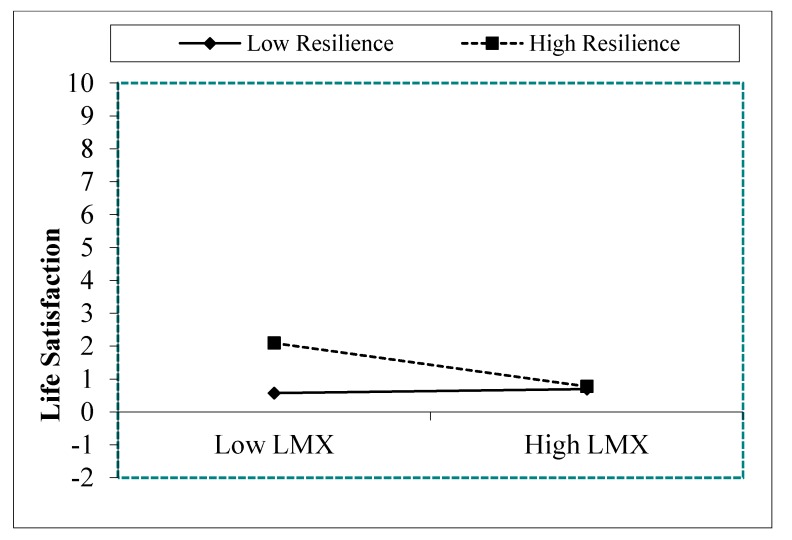
Interaction effects of resilience and LMX on life satisfaction Note. LMX = Leader Member Exchange.

Next, a new model was re-specified in the Chinese sample. In this model, resilience only moderated the relationship between LMX and life satisfaction. The re-specified model showed a satisfactory fit. The estimators (Table 3) indicate that all the estimated variables were statistically significant. As Figure 4 shows, higher levels of LMX were associated with low levels of life satisfaction when resilience was high (*t* = −0.05; *p* = 0.001), and low levels of LMX were associated with low levels of life satisfaction when resilience was low (*t* = 0.02; *p* = 0.001).

In summary, these results show that resilience plays a moderator role in the relationship between LMX and engagement and life satisfaction only in Spanish employees (hypothesis 3). In Chinese employees, resilience moderates the relationship between LMX and life satisfaction, but not between LMX and engagement. Life satisfaction was higher with when LMX was low and resilience was high. Moreover, the direction of the relationship between high LMX and life satisfaction and high resilience was contrary to our predictions.

## 4. Discussion

The aim of this study was to examine whether the quality of the relationship with the leader predicts engagement and life satisfaction, and whether resilience moderates this relationship in two cultural contexts (Spanish and Chinese). The results showed that LMX affects engagement and life satisfaction. Thus, when the leader has a high-quality relationship with subordinates, i.e., is closer, friendlier, more inclusive, and more communicative, this exchange makes employees feel more engaged at work and positively perceive other life domains (family, leisure activities, etc.). These findings support hypothesis 1 and are consistent with previous studies showing that high LMX was associated with high engagement, whereas low LMX was associated with low engagement [25,26,27]. Regarding life satisfaction, our results were consistent with Pytlovany [32], who indicates that work influences life satisfaction through the satisfaction of needs, and that supervisors help to contribute to or facilitate the satisfaction of these needs. Moreover, our study reveals that positive work life experiences spill over to other life domains (e.g., life satisfaction) [33,83]. With this study, we contribute by identifying some important mechanisms underlying satisfaction in work and non-work domains.

Likewise, the current study offers evidence that resilience moderates the relationship between LMX and engagement and life satisfaction. Our results highlight that the differences in the quality of the leader–subordinate relationship are buffered by resilience. Thus, resilience helps to understand how low LMX can be positively associated with engagement and life satisfaction. This result supports our hypothesis 2 and is consistent with Wang et al. [43], who state that personal and external factors together (e.g., resilience and LMX) lead to a positive way of facing adversities in employees.

In our study, the moderator effect of resilience varied across cultures. Resilience had a moderator role in the relationship between LMX and engagement and life satisfaction only in the Spanish sample. In the Chinese sample, resilience moderated the relationship between LMX and life satisfaction, but not between LMX and engagement. In the Spanish sample, our results show that having a high-quality relationship with the leader leads to more engagement and life satisfaction when resilience is high than when it is low. These results are congruent with previous research suggesting that employees’ behaviors and attitudes are contingent responses to the leader’s treatment in individualistic cultures, such as the Spanish culture [46,49,50]. Our results also agree with previous research asserting that work engagement is embraced more in individualistic cultures than in collectivistic cultures [27,60]. A collectivistic culture is more concerned with group achievements than with personal work goals [17].

In the Chinese sample, resilience only moderated the relationship between LMX and life satisfaction. In this culture, where there is greater respect for authority and high interdependence [49], having a low-quality relationship with the leader (low social resources) and high personal resources (resilience) increases employees’ life satisfaction. One possible explanation for this result is that resilience can be a compensation strategy for negative experiences at work, such as low LMX [33], and it can even have a protective effect during adversity [84]. In sum, in collectivistic cultures, resilience can be a personal resource for coping with unfavorable conditions.

### 4.1. Practical Implications

At the practical level, this study contributes to a better understanding of the relationship between leaders and subordinates operating in a global context. We studied LMX in two cultures (Chinese and Spanish) different from the North American culture. Thus, practitioners can gain knowledge about leadership based on practices developed without the North American mindset.

It is necessary to train leaders who operate in a globalized world. The competencies that make leaders effective in individualistic cultures can be different from competencies in collectivistic cultures [85]. In this regard, Avolio, Walumbwa, and Weber [86] suggested that leaders with high cultural intelligence (CQ), which refers to the ability to function and manage effectively in culturally diverse settings [87,88], are more able to manage the culturally diverse expectations of their followers. Thus, when leaders work extensively in international or cross-border settings, organizations should emphasize the development of cross-cultural capabilities, such as CQ.

Our findings also have implications for resilience studies. There is evidence that resilience can be taught and developed in the workplace [89]. Leaders could incorporate resilience management techniques to help employees face work and non-work demands [35].

### 4.2. Limitations and Future Research

This study has some potential limitations. First, self-report measures were used, which is justified by the nature of the variables considered in this study. These measures can cause common error bias. To mitigate common method variance, we used a different scale format and anchor point [70]. Future studies should use data from multiple sources. Second, it would be interesting to study the dynamics of the relationships between the variables of this research by using longitudinal designs. Third, our findings may have limited generalizability because we used a small or moderate sample, used incidental purposive sampling, and used only two cultures. However, we used bootstrapping methods to address potential small sample issues. In this sense, following Hickey et al. (2018), sample size “is only one element of a well-designed protocol”. Future research should replicate this study with specific samples (e.g., professors, police officers, etc.). This would help to generalize the results in a meta-analysis and know the characteristics of the groups according to the culture. On the other hand, Etikan et al. [69] state that this sampling method is useful for this type of study, but it is not truly representative of the population because of the subjective way the sample is chosen. Moreover, the sample represents a diverse sector of workers from Spain and China. In the same way, studying only two cultures could be a limitation related to the generalizability of the results. The extension of this research to additional individualistic and collectivistic countries is required before the results can be considered as widely applicable. On the other hand, González-Navarro, Olateju, Zurriaga, and Llinares-Insa [90] showed that LMX operated differently depending on the ownership of the company, and so future studies could analyze this as well. Finally, this study has not taken into account the nature of the professions in both cultures as a criterion for inclusion in the sample. Future research could compare similar professions in different cultures for a better understanding of cultural differences.

## 5. Conclusions

LMX is a social resource that can increase employee engagement and life satisfaction, and resilience is a personal resource that can modulate this relationship. Nevertheless, the moderator role of resilience varies across cultures. Our results indicate that resilience moderated the relationship between LMX and engagement and life satisfaction only in the Spanish sample. In the Chinese sample, resilience moderated the relationship between LMX and life satisfaction but not between LMX and engagement. This research supports the strong link between work and non-work domains (see [33,91]). Finally, our study contributes to a better understanding of the relationship between leaders and subordinates operating in a global context.

## Figures and Tables

**Table 1:** Descriptive statistics and bivariate correlations.

Variable	M	SD	1	2	3
**Total sample**					
1. Engagement	3.40	0.96			
2. LS	3.09	0.84	0.54 **		.
3. LMX	2.59	0.56	0.46 **	0.48 **	
4. Resilience	3.51	0.78	0.65 **	0.62 **	0.37 **
**Spanish employees**					
1. Engagement	3.65	1.19			
2. LS	3.56	0.85	0.46 **		
3. LMX	2.74	0.63	0.50 **	0.42 **	
4. Resilience	3.91	0.83	0.58 **	0.42 **	0.38 **
**Chinese employees**					
1. Engagement	3.11	0.43			
2. LS	2.52	0.30	0.77 **		
3. LMX	2.41	0.41	0.03	0.32 **	
4. Resilience	3.06	0.36	0.88 **	0.56 **	−0.20 *

Note: N = 224. * *p* < 0.05; ** *p* < 0.01; 1 = Engagement; 2 = Life Satisfaction; 3 = LMX; 4 = Resilience; LMX = Leader Member Exchange; LS = Life Satisfaction.

**Table 2:** Summary of structural equation modelling (SEM) analyses.

Model	χ^2^	d.f.	NFI	IFI	CFI	RMSEA
Total sample	4.17	1	0.99	0.99	0.99	0.10
Multi-group	59.51	2	0.92	0.92	0.92	0.32
Spanish employees	4.43	1	0.98	0.99	0.98	0.15
Chinese employees	4.43	1	0.98	0.99	0.98	0.15

Note. N_total sample_ *=* 225; N_Spanish employees_ = 150; N_Chinese employees_ = 127; χ^2^ = chi-square; d.f. = degrees of freedom; NFI = Normed Fit Index; IFI = Incremental Fit Index; CFI = Comparative Fit Index; RMSEA = Root Mean Square Error of Approximation.

**Table 3:** Standardized SEM effects.

Variables	Total Sample		Multi-Group Analysis				Spanish Employees		Chinese Employees	
			Spanish Employees		Chinese Employees					
	Est.	ρ	Est.	ρ	Est.	ρ	Est.	ρ	Est.	ρ
LMX-Engagement	0.21	0.01	0.26	0.01	0.05	0.27	0.26	0.01		
LMX-LS	0.22	0.01	0.15	0.02	0.09	0.05	0.15	0.02	0.16	0.05
Resilience-Engagement	0.49	0.01	0.48	0.01	0.82	0.01	0.48	0.01		
Resilience-LS	0.41	0.01	0.21	0.01	0.39	0.01	0.21	0.01	0.50	0.001
Resilience Moderator of LMX-Engagement	0.03	0.06	0.12	0.04	−0.10	0.2	0.12	0.04		
Resilience Moderator of LMX-LS	0.11	0.01	0.12	0.1	−0.13	0.01	0.12	0.01	−0.12	0.01

Note. N_Spanish employees_ = 150; N_Chinese employees_ = 127; Estimate = Beta value; LMX = Leader Member Exchange; LS = Life Satisfaction.
